# Mechanism of Osmolyte Stabilization–Destabilization of Proteins: Experimental Evidence

**DOI:** 10.1021/acs.jpcb.2c00281

**Published:** 2022-04-20

**Authors:** Marcin Stasiulewicz, Aneta Panuszko, Piotr Bruździak, Janusz Stangret

**Affiliations:** Department of Physical Chemistry, Gdańsk University of Technology, Narutowicza 11/12, Gdańsk 80-233, Poland

## Abstract

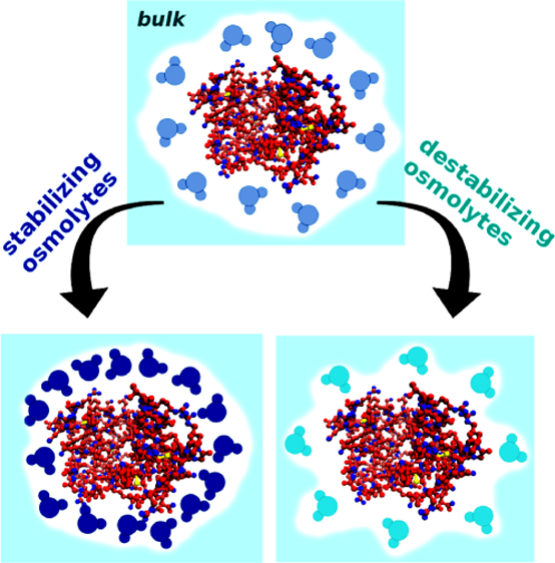

In this work, we investigated the influence of stabilizing (*N*,*N*,*N*-trimethylglycine) and destabilizing (urea) osmolytes on the hydration spheres of biomacromolecules in folded forms (*trpzip*-1 peptide and hen egg white lysozyme—*hewl*) and unfolded protein models (glycine—GLY and *N*-methylglycine—NMG) by means of infrared spectroscopy. GLY and NMG were clearly limited as minimal models for unfolded proteins and should be treated with caution. We isolated the spectral share of water changed simultaneously by the biomacromolecule/model molecule and the osmolyte, which allowed us to provide unambiguous experimental arguments for the mechanism of stabilization/destabilization of proteins by osmolytes. In the case of both types of osmolytes, the decisive factor determining the equilibrium folded/unfolded state of protein was the enthalpy effect exerted on the hydration spheres of proteins in both forms. In the case of stabilizing osmolytes, enthalpy was also favored by entropy, as the unfolded state of a protein was more entropically destabilized than the folded state.

## Introduction

Osmolytes, a group of small organic molecules, can alter the stability of proteins.^1,2^ They can be divided into two subgroups, namely, stabilizers and destabilizers (or denaturants), according to their influence on proteins.^3,4^ The mechanism of the influence has been the focus of many studies,^5−14^ yet there is no general agreement on the mode of action of osmolytes. One of the main theories—“the osmophobic theory”—states that the interactions between stabilizing osmolytes and the peptide backbone of both the native and denatured states are unfavorable. As a result, stabilizing osmolytes are excluded from the vicinity of proteins.^8,12,15−23^ The surface of proteins is larger in the case of unfolded forms; thus, the equilibrium of the folding reaction is shifted toward the native state due to the entropy effect associated with the exclusion of the volume available for the osmolyte molecules. In turn, the driving force of protein unfolding by destabilizing osmolytes is the favorable enthalpy change, which results from a higher number of active binding centers accessible to denaturants in unfolded proteins.^12,24^ Another hypothesis states that the stabilization or destabilization of proteins is the result of an indirect influence of osmolytes arising from the change in water properties in their solutions.^6,10,22,25−27^ Some research teams indicate that the real mechanism can be a mix of both direct and indirect mechanisms.^6,28^

Our previous results point out the crucial role of the hydration water of osmolytes and its similarity or dissimilarity in the hydration water of proteins.^29^ It has been demonstrated that stabilizing osmolytes [trimethylamine *N*-oxide, glycine (GLY) and its *N*-methyl derivatives, and amino acids] show a very similar type of hydration in terms of energy and hydrogen bond length distribution, which corresponds well to the type of protein hydration.^29,30^ The only exception is taurine.^31^ In the case of both proteins and stabilizing osmolytes, hydrogen bonds in the hydration water are significantly strengthened in relation to the pure water. Conversely, destabilizing osmolytes (urea and its alkyl derivatives) also show a similar type of hydration to each other but differ significantly from that of the protein. The hydration water around this group of osmolytes resembles pure water but with a narrower probability distribution of the hydrogen bond lengths, that is, an increased population of water molecules with properties most likely to be found in pure water. Many other experimental^23,27,32−44^ and theoretical^27,36,39,42−52^ studies on the effect of osmolytes on water have been published to date.

It should be emphasized that, at this point, knowledge of the influence of osmolytes on the properties of pure water is a separate issue with regard to their influence on the hydration sphere of proteins. This statement is confirmed by several findings. The most spectacular ones concern the difference in the influence of urea and taurine on the state of pure water and on the state of water in the hydration sphere of the reference molecules. It is well known that urea changes the properties of pure water to a very small extent,^32−34,39,44,46,52^ while it significantly weakens the hydrogen bonds of the hydration water of molecules modeling the properties of the surface of proteins in a folded form.^53^ Taurine, conversely, strongly weakens the hydrogen bonds of water in its environment^31^ and simultaneously stabilizes the folded form of proteins.^31,54^ Therefore, in the context of the problem studied in this paper, there is insufficient knowledge of the influence of osmolytes on the surrounding water, and it is necessary to understand this influence on the hydration sphere of the protein in its folded and unfolded forms. Information on this subject is very incomplete and scarce in the literature, even in those studies performed using theoretical methods.^53^

In light of the information provided earlier regarding the similarity of hydration of previously investigated stabilizing and destabilizing osmolytes, in this work, we selected *N*, *N*, *N*-trimethylglycine (TMG, betaine) as a representative stabilizer and urea as a representative destabilizing osmolyte and examined their influence on the folded form of real biomacromolecules: *trpzip*-1 peptide and hen egg white lysozyme (*hewl*). *Trpzip*-1 is a simple β-hairpin peptide with a well-defined sequence and a stable secondary structure in solution. Lysozyme is a small globular protein with a well-known structure and is often used as a model protein for studying the protein-folding process. It should be emphasized that at the temperature of the experiment, 25 °C, both biomacromolecules are virtually only in their native folded states, even in the most concentrated urea solution used in our studies. Measurements of an appropriate influence of osmolytes on the unfolded form of these biomacromolecules encounter experimental difficulties because these compounds, after thermal denaturation and cooling to 25 °C, show a highly distorted, aggregated, or only partially renatured structure. These features force the use of model molecules in place of the practically unattainable unfolded forms of the studied proteins at room temperature. When selecting these models, we were guided by the following arguments. We have numerous theoretical and experimental premises to support that the folded form of a protein has a hydration sphere characterized by stronger hydrogen bonds than the hydration sphere of its unfolded form because the surface of the folded form of a protein is characterized by its immediate proximity to hydrophilic centers and hydrophobic regions, in contrast to the surface of the chain of the unfolded form of the protein. Previously, we were able to show, using the hydration of NMA (*N*-methylacetamide)^55^ and DMSO (dimethylsulfoxide)^56^ model molecules, that such a direct proximity favors the formation of a strong clathrate-like water structure around closely located hydrophobic groups. However, such an enhancement was possible only if the interaction of water with polar groups was stronger than that between bulk water molecules. This cooperativity of hydrogen bonds of water molecules, that is, between those interacting with the hydrophilic centers and water molecules at the hydrophobic surface of the protein, results in anchoring the shared network of water hydrogen bonds on the hydrophilic group and its dynamic stabilization. In this work, we used GLY and *N*-methylglycine (NMG) as models of unfolded peptides or protein fragments. GLY does not have a typical hydrophobic group—our observation suggests that the CH_2_ group in the surroundings of polar groups, as in the case of amino acids, is not a sufficient hydrophobic center to organize water molecules in a manner characteristic of hydrophobic hydration. Other studies support this perspective.^57^ In the case of NMG, the hydrophobic group is not directly adjacent to the polar group. The adopted models intended to correspond to fragments of the unfolded protein clearly have significant limitations due to the different chemical structures with respect to the protein molecules. It should also be noted that the hydrophobic effects depend on the solute size^58^ and therefore may be different for small solutes (i.e., model molecules) and large solutes (i.e., proteins). In turn, the size of the molecules may affect their electronic properties, which play an important role in the way osmolytes interact with proteins.^59^ However, the selected model molecules have the abovementioned important feature, which, in our opinion, may qualify them for the assigned role.

The influence of selected representatives of stabilizing and destabilizing osmolytes on the hydration sphere of the selected peptide and protein and model molecules can be studied as a function of the osmolyte concentration by means of Fourier transform infrared (FTIR) spectroscopy and a recently developed method of data analysis.^53^ This method allowed us to isolate the part of the water that is simultaneously modified by the biomacromolecule (or model molecule) and the osmolyte. Data obtained from this analysis are crucial for understanding the mechanism of the role of osmolytes in the stabilization/destabilization of biological systems.

## Methods

### Chemicals and Solutions

GLY (Aldrich, Darmstadt, Germany ≥99%) and NMG (Fluka, Steinheim, Germany, ≥99%) were used as model molecules. The *Trpzip*-1 peptide (SWTWEGNKWTWK, Lipopharm.pl, Zblewo, Poland) and the hen egg white lysozyme (*hewl*, 129 amino acid residues, Fluka, Steinheim, Germany) were used as biomacromolecules.

TMG (Alfa Aesar, Karlsruhe, Germany, 98%) and urea (Aldrich, Darmstadt, Germany, 99.5%) were used as osmolytes.

Two series of solutions for each system were prepared for the measurements—semiheavy water (HDO) solutions and reference H_2_O solutions. Each series consisted of model solutes or biomacromolecules at a constant molal concentration and varying concentrations of osmolyte. Molalities of GLY (ca. 70 mg mL^–1^) or NMG (ca. 83 mg mL^–1^) in solution were approximately 1 mol kg^–1^, and molalities of *trpzip*-1 and *hewl* were ca. 0.035 mol kg^–1^ (ca. 53 mg mL^–1^) and 0.008 mol kg^–1^ (ca. 102 mg mL^–1^), respectively. Samples of the HDO series were prepared by adding D_2_O to each of them at 4% of the total mass of water in solutions. Equal molar amounts of water were added to samples of the reference series.

### FTIR Measurements

A Nicolet 8700 spectrometer (Thermo Electron Co., Waltham, Massachusetts, US) was used to register the FTIR spectra of solutions at 25.0 ± 0.1 °C. For measurements, a liquid transmission cell (model A145, Bruker Optics, Billerica, Massachusetts, US) with CaF_2_ windows separated by PTFE spacers, a KBr beamsplitter, a DTGS TEC detector, and the EverGlo IR source was used. The path length was equal to 0.029 mm, as determined interferometrically. Each spectrum consisted of an average of 256 independent scans with a selected resolution of 4 cm^–1^. The spectrometer was purged with dry nitrogen during the measurement. Spectra were analyzed using commercial software: GRAMS/32 4.01 (Galactic Industries Corporation, Salem, NH, USA) and RAZOR (Spectrum Square Associates, Inc, Ithaca, NY, USA.) run under GRAMS/32. The parameters of spectral bands (gravity centers, position, etc.) were calculated using GRAMS/AI version 9.3 (Thermo Fisher Scientific Inc).

### Spectral Data Analysis

The OD stretching vibration bands of HDO are used to probe the state of water in solution. The applied method of HDO spectral analysis is based on the quantitative method of difference spectra.^60,61^ In its simplest version, it leads to the isolation of the spectral fraction of those water molecules that are affected by the solute (i.e., are under its influence in the spectroscopic sense).

The boundary cases occurring in a solution consisting of two different solutes are illustrated in Figure S1 in the Supporting Information. The details of the spectral data method analysis in systems with two solutes and details of the cases in Figure S1 are described in the Supporting Information and in ref (53). The analysis procedure includes several steps. First, the affected water spectrum in an aqueous solution containing only one solute, a model molecule, a biomacromolecule (m) or osmolyte (o) and the corresponding *N* number, that is, the number of moles of water molecules affected by one mole of the solute, are determined. The second step involves isolation of the affected water spectrum in the solution containing two solutes (model molecule/biomacromolecule and osmolyte): the so-called “experimental” spectrum of affected water and the average number of moles of affected water molecules by one mole of solutes m and o taken together (i.e., *n*_m_ + *n*_o_ = 1 mol), *N*_e_. Next, the so-called “synthetic” spectrum of affected water is constructed from the spectra of affected water obtained for pure components: model molecule/biomacromolecule and osmolyte. This spectrum corresponds to the average number of moles of water molecules affected by one mole of solute taken together, *N*_s_. The “synthetic” affected spectrum corresponds to the hypothetical situation, where hydration spheres of both solutes are isolated in solution and any interactions between them are absent. The final step is the isolation of the spectrum of water affected simultaneously by both solutes, the so-called “double” affected water spectrum (Figures S7 and S8 in the Supporting Information). For this purpose, the “synthetic” water spectrum (Figures S5 and S6 in the Supporting Information) is subtracted from the “experimental” water spectrum (Figures S3 and S4 in the Supporting Information). Details for extracting the “double” affected water spectra are described in the Supporting Information. To facilitate the comparison of the number of affected water molecules corresponding to the “experimental” (*N*_e_) and “synthetic” (*N*_s_) affected spectra, the values of the affected water molecules were transformed into the *N*_p_ functions: *N*_pe_ = *N*_e_·(β + 1) or *N*_ps_ = *N*_s_·(β + 1). These functions present the situation for the set consisting of 1 mol of a model molecule/biomacromolecule and β moles of an osmolyte (where β is a molar ratio of the osmolyte to the model molecule/biomacromolecule in solution). The differences in *N*_p_ for “experimental” and “synthetic” affected water spectra for studied systems are presented in Tables S1 and S2 in the Supporting Information.

The interpretation of the spectral results is based on the Badger–Bauer rule,^62^ according to which the energy of hydrogen bonds changes proportionally to the shift of the OD (OH) band position. Stronger hydrogen bonds correspond to lower wavenumbers of the OD band position. The value of the positions of the OD band gravity center (ν^g^) is a measure of the average hydrogen bond energy, while the band position at its maximum corresponds to the most likely energy.

The structural state of biomacromolecule or model molecule hydration water under the influence of the osmolyte can be characterized by the interatomic O···O distance distribution function, *P*(*R*_OO_). For this purpose, transformation of the spectral contours of the “double” affected water and water affected by the biomacromolecule/model molecule was performed according to the procedure described in refs (63) and (61) using the empirical function from ref (64).

## Results

### Influence of Osmolytes on the Hydration Spheres of Folded Forms of Biomacromolecules

**Figure 1:**
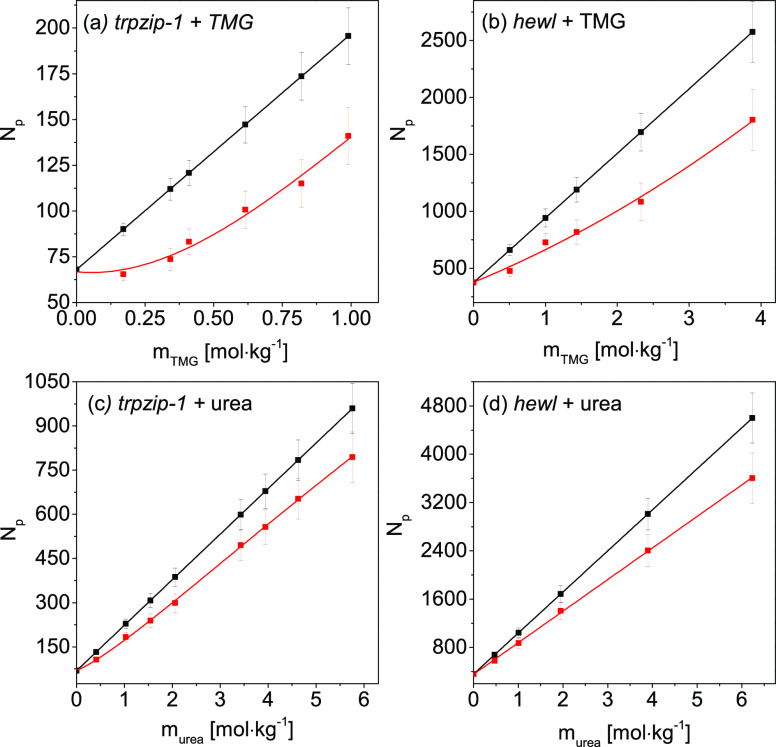
Numbers of affected water molecules, *N*_p_, obtained for “experimental” (red squares) and “synthetic” affected water spectra (black squares) as a function of molality of the osmolyte for (a) *trpzip*-1 + TMG, (b) *hewl* + TMG, (c) *trpzip*-1 + urea, and (d) *hewl* + urea systems. See the Supporting Information for details regarding the error bar determination.

The “experimental” and “synthetic” spectra of affected water for biomacromolecules in the presence of osmolytes (*trpzip*-1 + TMG, *hewl* + TMG, *trpzip*-1 + urea, and *hewl* + urea) are shown in Figures S3 and S5. The numbers of affected water molecules (*N*_p_) determined for these spectra as a function of osmolyte molality are presented in Figure 1. In each case, *N*_p_ for the “experimental” spectra is smaller than *N*_p_ for the “synthetic” spectra. This observation means that some water molecules are shared between the hydration spheres of both solutes due to overlapping of these spheres (see Figure S1d).

**Figure 2:**
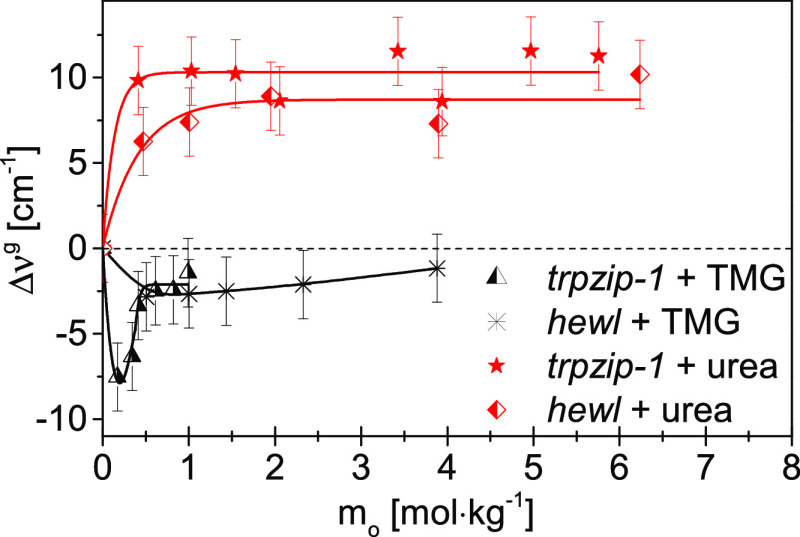
Differences in the values of the gravity center of bands (a measure of the average hydrogen bond energy of water molecules) between the “double” affected water and the water affected by the model biomacromolecule (Δν^g^ = ν_double_^g^ – ν_m_^g^) as a function of osmolyte molality (*m*_o_). Red lines indicate systems involving urea. Black lines indicate systems involving TMG.

**Figure 3:**
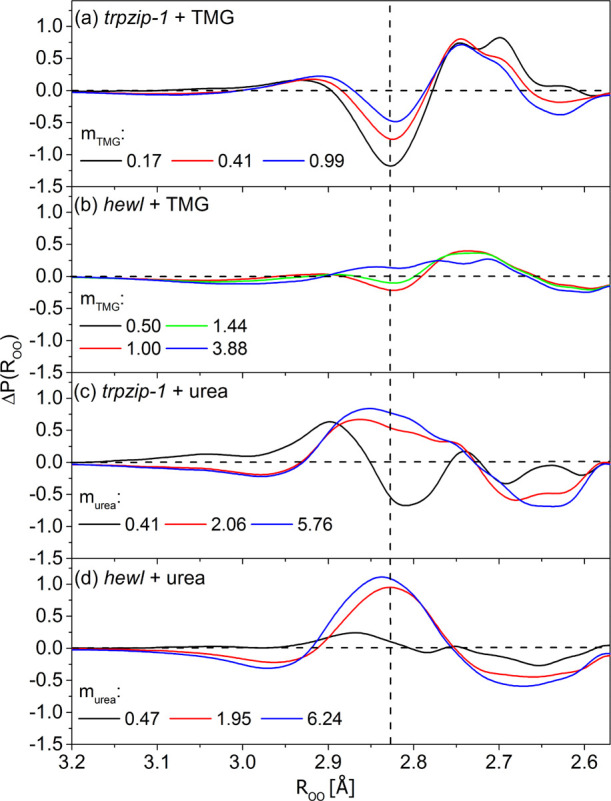
Differences between the interatomic O···O distance distribution function, Δ*P*(*R*_OO_) = *P*(*R*_OO_)_double_ – *P*(*R*_OO_)_macromolecule_, as a function of osmolyte molality (*m*) [mol kg^–1^] in (a) *trpzip*-1 + TMG, (b) *hewl* + TMG, (c) *trpzip*-1 + urea, and (d) *hewl* + urea systems. The vertical dashed line corresponds to the most likely O···O distance in pure water (2.83 ± 0.01 Å).

To compare the effect of both osmolytes on the hydration spheres of biomacromolecules in terms of the hydrogen bond energy of water, differences in the values of the gravity center of bands between the “double” affected water (Figure S7) and the water affected solely by the biomacromolecule (Δν^g^) as a function of the osmolyte molality were calculated and are presented in Figure 2. Negative Δν^g^ values indicate stronger hydrogen bonds in the “double” affected water than in the water affected only by a biomacromolecule, whereas positive values of Δν^g^ mean that water hydrogen bonds in the “double” affected water are weaker. For biomacromolecule–TMG systems, the enhancement of the energy of hydrogen bonds in the hydration spheres of biomacromolecules (negative Δν^g^ values) is clearly visible in the whole range of stabilizing osmolyte molalities. The increase in the energy of hydrogen bonds is more pronounced at low molalities of TMG in the *trpzip*-1 + TMG system. A possible explanation for this phenomenon is the ligand-like behavior of TMG.^65^ At low molality, TMG accumulates near specific interaction centers on the protein/peptide surface; therefore, it strongly influences water molecules shared with the biomacromolecule. At some specific molality, those interaction sites are saturated. The rest of the less specifically located TMG molecules enhance the less-effective water hydrogen bond energy. This effect is also visible in Figure 3a as a population of very strong hydrogen bonds (at 2.70 Å) at the lowest TMG molality. It shifts toward a slightly weaker interaction (but still strong) at higher molalities of TMG. However, the hydrogen bonds in the hydration spheres of biomacromolecules are weakened in the presence of destabilizing osmolyte—urea (positive Δν^g^ values).

Next, Δ*P*(*R*_OO_) functions (Figure 3) are obtained by subtracting the O···O distance distribution function for water affected by the biomacromolecule, *P*(*R*_OO_)_macromolecule_, from an analogous distance distribution function for “double” affected water, *P*(*R*_OO_)_double_. This operation enabled us to visualize the influence of osmolytes on the hydration sphere of *trpzip*-1 and *hewl* in the context of intermolecular distance. Figure 3 shows that the presence of TMG in the *trpzip*-1 and *hewl* solutions increases the population of strong hydrogen bonds (positive Δ*P*(*R*_OO_) values), with an O···O length of approximately 2.74 Å (which corresponds to the distances in ice^66^). This increase is accompanied by a reduction in the population of water molecules at distances of approximately 2.83 Å (negative Δ*P*(*R*_OO_) values), which corresponds to the most likely distance in pure water. The only exception is visible in the *hewl* + TMG system at the highest molality of TMG, where a slight increase in this population is observed. It is worth noting that the differences Δ*P*(*R*_OO_) obtained for the *hewl* + TMG system are smaller than those for the *trpzip*-1 system, indicating that TMG has a reduced effect on the hydration sphere of *hewl* than on that of *trpzip*-1.

The effect of urea on the hydration sphere of *trpzip*-1 and *hewl* is opposite. In the case of both biomacromolecules, at the lowest molality of urea, an increase in weak and long hydrogen bonds (2.87–2.90 Å) and a decrease in strong and short hydrogen bonds are observed. At higher urea molalities, a distinct population of hydrogen bonds with distances equal to and longer than the most likely distance in pure water (2.83 Å) emerges. Moreover, in contrast to TMG, urea influences the hydration spheres of both biomacromolecules to a similar extent.

### Influence of Osmolytes on the Hydration Spheres of Model Molecules in Relation to the Fragments of Unfolded Forms of Proteins

**Figure 4:**
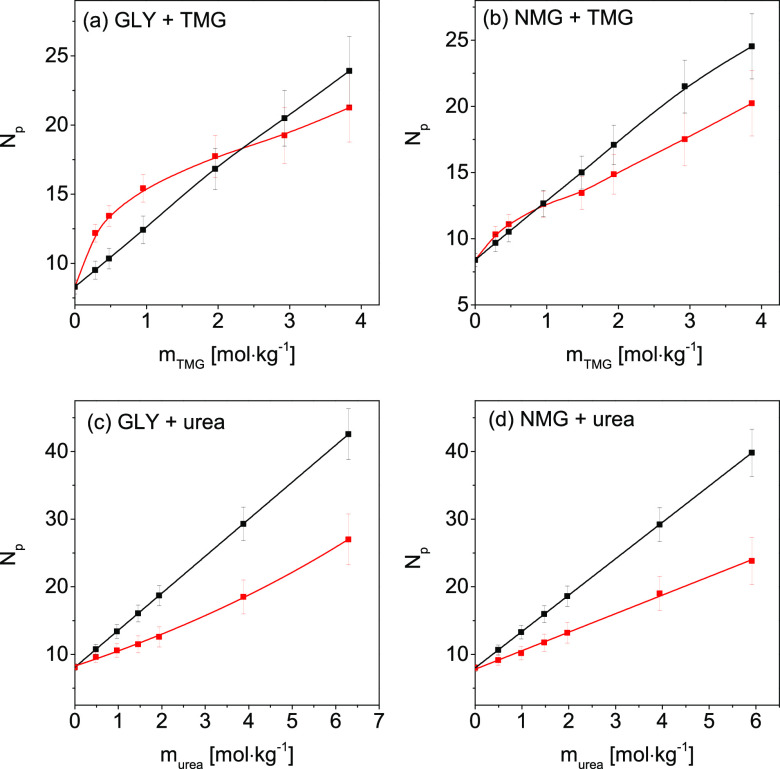
Numbers of affected water molecules, *N*_p_, obtained for “experimental” (red squares) and “synthetic” affected water spectra (black squares) as a function of molality of the osmolyte for (a) GLY + TMG, (b) NMG + TMG, (c) GLY + urea, and (d) NMG + urea systems. See the Supporting Information for details regarding the error bar determination.

The “experimental” and “synthetic” spectra of affected water for model molecules in the presence of osmolytes (GLY + TMG, NMG + TMG, GLY + urea, and NMG + urea) are presented in Figures S4 and S6. Figure 4 shows the numbers of affected water molecules (*N*_p_) obtained for these spectra as a function of osmolyte molality. As shown in Figure 4a,b, the *N*_p_ values corresponding to the “experimental” spectra are higher than those of the “synthetic” spectra at molalities lower than 2.4 and 1 mol kg^–1^ for GLY + TMG and NMG + TMG systems, respectively. This finding indicates that additional water molecules are affected, that is, the cross-linking water (see Figure S1b). Conversely, at the higher molalities of stabilizing osmolytes, the *N*_p_ values for the “experimental” spectra are lower than those for the “synthetic” spectra. This result indicates the existence of shared affected water, that is, hydration sphere overlap (see Figure S1d). In the case of systems with urea (Figure 4c,d), the number of affected water molecules (*N*_p_) for the “experimental” spectra is lower than that for the “synthetic” spectra in the whole range of studied molalities.

**Figure 5:**
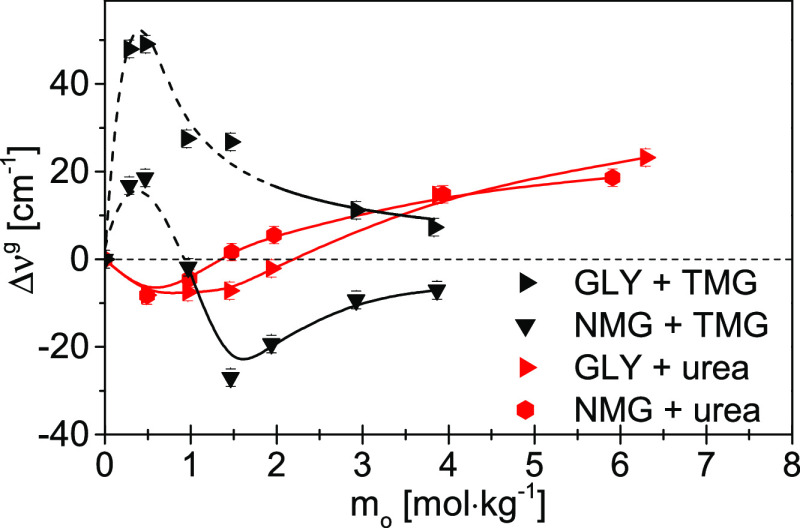
Differences in the values of the gravity center of bands (a measure of the average hydrogen bond energy of water molecules) between the “double” affected water and the water affected by the model molecule (Δν^g^ = ν_double_^g^ – ν_m_^g^) as a function of osmolyte molality (*m*_o_). Red lines indicate systems involving urea. Black lines indicate systems involving TMG.

The influence of TMG and urea on the hydration spheres of model molecules in the energetic aspect of Δν^g^ is shown in Figure 5. The “double” affected water spectra obtained for these systems are shown in Figure S8. The analysis of Δν^g^ indicates that H-bonds in the hydration sphere of GLY are weakened in the presence of TMG (positive values of Δν^g^) when compared to water affected only by GLY. This situation occurs in the entire range of TMG molalities, both in the case of water linking the hydration spheres of two solutes (Figure S1b) and shared affected water (Figure S1d). In the NMG + TMG system at molalities below 1 mol kg^–1^, the energy of hydrogen bonds of “double” affected water is lower relative to NMG-affected water (positive values of Δν^g^), while at higher molalities of TMG it is higher (negative values of Δν^g^). Moreover, the enhancing effect of TMG on the NMG hydration sphere and weakening on the GLY hydration sphere decreases with the increasing molality of TMG.

The impact of urea on the hydration spheres of both model molecules is similar: hydrogen bonds in their hydration spheres are strengthened (negative Δν^g^) at molalities below 2.3 or 1.5 mol kg^–1^ for GLY + urea and NMG + urea systems, respectively. Above these molality values, weakening of the hydrogen bonds of water around the model molecules becomes noticeable, manifested by positive values of Δν^g^. This phenomenon is definitely different from the one that occurs in the case of hydration spheres of biomacromolecules in the folded form studied in this work but also from simple protein modeling solutes, such as NMA and DMSO,^53^ where the energy of hydrogen bonds is weakened, even at the lowest urea concentrations. It seems most likely that urea interacts initially via its carbonyl group with those water molecules that form hydrogen bonds with the hydrogen atoms of the amino group of GLY (three such interactions are possible) or amino group of NMG (two such interactions are possible). Further impact of urea relies on hydrogen bonds via the amino groups of the molecule, which results in a reduction in their strength. As is known,^46,67,68^ hydrogen bonds between the −NH_2_ group of urea and water are weak and at least significantly weaker than interactions of the water–water type. Only urea interactions via the carbonyl group are stronger than water–water interactions.^46,67,68^ Direct interactions of urea with model molecules, resulting in the removal of water molecules from their strong centers of interaction, are also a possible reason for weakening of the hydrogen bonds of hydration water at higher urea molalities.

**Figure 6:**
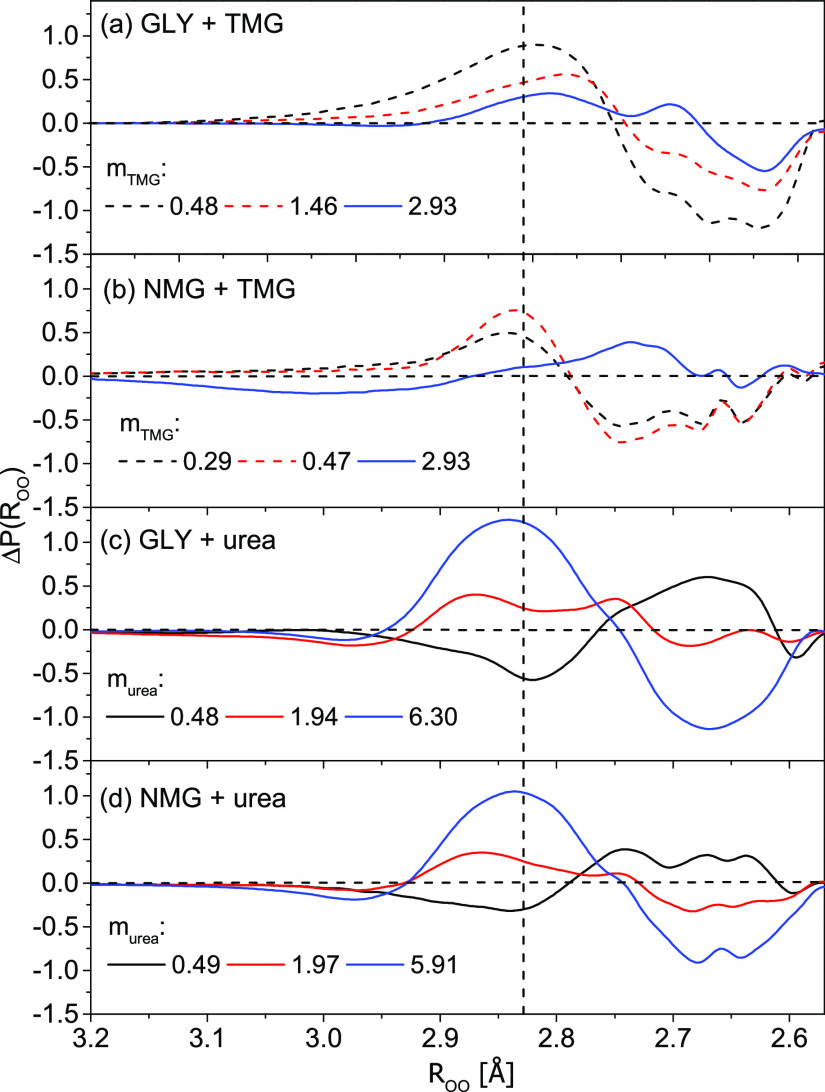
Differences between the interatomic O···O distance distribution function, Δ*P*(*R*_OO_) = *P*(*R*_OO_)_double_ – *P*(*R*_OO_)_model_, as a function of osmolyte molality (*m*) [mol kg^–1^] in systems of (a) GLY + TMG, (b) NMG + TMG, (c) GLY + urea, and (d) NMG + urea. The dashed line indicates cross-linking water, while the solid line indicates shared water. The vertical dashed line corresponds to the most likely O···O distance in pure water (2.83 ± 0.01 Å).

More information on the structural state of hydrogen bonds of water under the simultaneous influence of the model molecule and osmolyte (i.e., “double” affected water) was obtained on the basis of differences in the distribution of distances between “double” affected water and water affected only by the model molecule, Δ*P*(*R*_OO_), (see Figure 6).

In the GLY + TMG (Figure 6a) and NMG + TMG (Figure 6b) systems, at the lowest TMG molalities, the population of moderate and weak hydrogen bonds (2.80–2.95 Å) increases, and simultaneously, the population of strong hydrogen bonds (2.75 Å) decreases relative to water affected by the model molecule (GLY or NMG). It is worth noting that for these systems, at these molalities, cross-linking “double” affected water occurs. For the highest molality of TMG (2.93 mol kg^–1^), a smaller increase in the water hydrogen bond population with O···O distances at approximately 2.83 Å takes place (in the NMG + TMG system, the contribution of these bonds is insignificant), and an increase in the population of strong hydrogen bonds can be noticed. Δ*P*(*R*_OO_) obtained for this molality corresponds to the situation in which the hydration spheres of both molecules overlap with each other (i.e., shared “double” affected water).

The effect of urea on the hydration spheres of GLY and NMG is similar (Figure 6c,d). At the lowest urea molality (approx. 0.5 mol kg^–1^), the population of hydrogen bonds between water molecules with O···O distances of approximately 2.83 Å decreases, while the population of very strong hydrogen bonds (2.75 Å) increases (compared with water affected by model molecules: GLY or NMG). With the increase in the urea concentration, the contribution of medium and weak hydrogen bonds increases, whereas the contribution of very strong hydrogen bonds decreases. At the highest concentration of urea (6.30 and 5.91 mol kg^–1^ for GLY + TMG oraz NMG + TMG systems, respectively), only a significant population of medium and weak hydrogen bonds can be observed.

## Discussion

The hypothesis of the indirect influence of osmolytes on protein stability has led researchers to investigate the influence of osmolytes on the structure and properties of water in their surroundings.^23,27,33,35,36,38−40,42−44,46−48,51,52^ A popular assumption was that kosmotropic solutes (structure-makers) should stabilize protein, while chaotropic (structure-breakers) solutes should destabilize them.^69,70^ However, results of studies on the influence of osmolytes on water are frequently contradictory,^38−40,43−46,48,49,69,71^ due to the use of different research methods and different parameters for water characterization.^72^ Some researchers indicate that it is impossible to connect the structure-making/breaking properties of osmolytes with their stabilization/destabilization attributes.^43,52,73^ However, an important factor is not taken into account in such studies. Namely, the influence of osmolytes on the hydration layer of peptides is rarely described or addressed.

In our studies, a short *trpzip*-1 peptide and *hewl* protein were used to study the hydration of a real-life biomacromolecule in its folded form in osmolyte solutions. As these molecules in their unfolded form were practically unavailable at room temperature, we used models (GLY and NMG) of unfolded protein fragments in our research. TMG and urea were used as representatives of stabilizing and destabilizing osmolytes, respectively. In our work, we used an experimental method that provided us with the characteristics of hydration water in terms of the energy and length of hydrogen bonds. With this in mind, we can discuss the mechanism of stabilization/destabilization of proteins by osmolytes.

TMG strengthens the hydration sphere of biomacromolecules in their folded form, while urea weakens it (Figure 2). The effect is very similar to that previously observed for the simple model molecules NMA and DMSO.^53^ Thus, NMA and DMSO appeared to be adequately selected models of the surface features of folded proteins.

When TMG is considered, it should be noted that hydrogen bonds in the hydration spheres of TMG^42,74^ and proteins^30,75,76^ are stronger than those in pure water. The interaction between these enhanced hydration spheres introduces an additional strengthening due to the cooperative nature of hydrogen bonds. This general description is based on the averaged characteristics of the hydrogen bonds in the hydration spheres. Given the diverse nature of the protein surface, such enhancing interactions are only likely at areas, where they have a chance to occur, that is, where close proximity to polar and nonpolar groups takes place.

Conversely, although urea is a weak structure maker in aqueous solution,^46,52,67^ it significantly weakens the hydration spheres of biomacromolecules. This result confirms our previous statement that urea disturbs the cooperativity of the hydrogen bonds of water hydrating the adjacent hydrophilic and hydrophobic regions on the surface of the biomacromolecule.^53^ Such proximity of both group types is much more likely in the case of protein in the folded form than unfolded ones; thus, the presence of the aforementioned cooperativity is more typical of the former case. We still need to stress the ability of urea to interact directly with the surface of protein, especially in areas where hydrophobic groups are present,^53,65,77−79^ resulting in the partial release of hydration water molecules.^80^ This phenomenon explains the interruption of the continuity of the hydrogen bond network of water molecules hydrating polar and nonpolar areas that are in close proximity. In this context, we find justification for the weakening of the hydration sphere of folded proteins by urea, despite its poorly enhancing effect on water. Accordingly, urea destabilizes the native form of the protein and peptide in the entire range of osmolyte concentrations. Other studies on the native protein (ferrocytochrome *c*)^81^ and the poly(*N*-isopropylacrylamide) brushes^82^ have shown that urea can act as a stabilizer at low concentrations, while at higher concentrations, it acts as a denaturant.

The examined osmolytes influence the hydration spheres of the unfolded fragment models (GLY, NMG) to a different extent than the hydration spheres of real biomacromolecules (Figures 2 and 5). TMG is excluded from the hydration spheres of models at low molalities. This phenomenon is confirmed with the incorporation of additional water molecules, taken from the bulk, in the vicinity between hydration spheres of this osmolyte and a model. These water molecules cross-link their hydration spheres but also indicate that TMG, with its hydration layer, avoids any direct contact with model molecules. Such behavior supports the entropic mechanism of protein stabilization.^19,83^ In the case of the unfolded form of proteins, the excluded volume for the osmolyte should be larger when compared to the case of the folded form,^84^ which leads to a shift of protein equilibrium toward the folded form. It should be noted that the exclusion effect is not observed in the case of the currently studied biomacromolecules; however, it was observed in the case of NMA, the model molecule of the folded form of protein, at low TMG molality.^53^ Excess water molecules cross-linking the GLY (or NMG) and TMG hydration spheres are characterized by weaker hydrogen bonds when compared to those in the hydration sphere of these models. As a result, TMG lowers the hydrogen bond energy of the GLY hydration layer for the entire osmolyte molality range investigated (although, above the molality of ca. 2 mol kg^–1^, the interaction changes its character), and in the case of NMG to a molality of ca. 1 mol kg^–1^ (Figure 5). These findings indicate that the hydration sphere of the protein in its folded form is energetically stabilized by the presence of TMG when compared to the case of unfolded models. At higher molalities, in the case of NMG, enhancement of the hydrogen bonds is observed. However, the enhancement is weaker when the TMG contributions in the solution increase (Figure 5). The latter result indicates the appearance of a specific strengthened molecular structure composed of water and both solutes, which depends on the solution composition. Summarizing the influence of TMG, two mechanisms of stabilization of the protein folded form are visible: energetic stabilization of the hydration sphere of the folded form^85−87^ and stronger entropic destabilization of the unfolded form.

Urea initially strengthens the hydration spheres of GLY and NMG (Figure 5). This behavior is clearly distant from its effect on the hydration water of *tripzip*-1 and *hewl*, where hydrogen bonds become weaker at the lowest urea molality (Figure 2). In the case of GLY and NMG, at higher urea molalities, a weakening of the hydrogen bonds of the hydrating water is observed, which may be the result of a direct interaction of osmolyte and the model molecules.

The obtained results clearly indicate that the energetic effect of urea on the hydration sphere of the folded and unfolded forms of protein determines the shifting of the denaturation equilibrium toward the unfolded structure. This effect seems to be associated with the direct interaction of urea molecules with the protein surface in the folded state.

## Conclusions

In this work, we investigated the impact of stabilizing (TMG) and destabilizing (urea) osmolytes on hydration spheres of biomacromolecules in folded forms (*trpzip*-1 peptide and *hewl*) and hydration spheres of models of unfolded peptide and protein fragments (GLY and NMG). The results obtained for GLY and NMG, as minimal models of the unfolded state of a protein, must be treated with caution due to their significant limitations. We provide information on the structural and energetic states of those water molecules that are simultaneously under the influence of both solutes: the biomacromolecule/model molecule and the osmolyte. Our findings allow us to propose a mechanism for the stabilization/destabilization of proteins by osmolytes.

An important feature of biomacromolecules in the folded state is the close proximity of the superficial hydrophilic and hydrophobic groups. Water molecules, those involved in hydrogen bonding with the hydrophilic group and those interacting with each other around the hydrophobic groups, cooperate in the formation of a strong hydration sphere around the biomacromolecule. The addition of TMG, with its enhanced hydration sphere, to the biomacromolecule solution causes additional strengthening of the hydration sphere around the protein or peptide. Conversely, the presence of urea in the solution destroys the water hydrogen bond network between the polar and nonpolar groups, and as a result, the water hydrogen bonds in the hydration sphere of the biomacromolecule are weakened. Accordingly, both osmolytes exert an enthalpy effect on the hydration sphere of the protein in the folded form: a stabilizing enthalpy effect in the presence of a stabilizing osmolyte and a destabilizing enthalpy effect in the presence of a destabilizing osmolyte.

However, both osmolytes have a different effect on the hydration spheres of the unfolded protein models (GLY and NMG), where hydrophilic and hydrophobic groups are more distant from each other or one of them is absent (GLY). Stabilizing osmolytes avoids direct contact with the unfolded protein, which supports the entropic mechanism of protein stabilization by osmolytes. In the presence of the destabilizing osmolyte, enthalpy stabilization of the hydration sphere of the unfolded protein occurs.

Considering the above observations, the most important factor determining the effect of osmolytes on proteins is the enthalpy effect exerted on their hydration spheres, both in the folded and unfolded forms. Furthermore, in the case of a stabilizing osmolyte, the entropy effect is also supportive because entropic destabilization of the unfolded form occurs.

## Supporting Information Available

Methods used for the data analysis; solute-affected water spectra; difference in the number of affected water molecules obtained for “experimental” and “synthetic” affected water spectra; and pH changes as a function of osmolyte molality for the biomacromolecule + osmolyte system (PDF)
