# Supplementary material for: Mechanism of Osmolyte Stabilization–Destabilization of Proteins: Experimental Evidence
Source: J Phys Chem B. 2022 Apr 20;126(16):2990–9. doi: 10.1021/acs.jpcb.2c00281 (PMC9059127; doi:10.1021/acs.jpcb.2c00281)
Supplement: Supplementary file 1 — jp2c00281_si_001.pdf [file jp2c00281_si_001.pdf]

## Supporting Information for Publication

# Mechanism of Osmolyte Stabilization–Destabilization of Proteins. Experimental Evidence

M. Stasiulewicz, A. Panuszko, P. Bruździak, and J. Stangret*

*Department of Physical Chemistry, Chemical Faculty, Gdańsk University of Technology,*
*Narutowicza 11-12, 80-233 Gdańsk, Poland*
email: janusz.stangret@pg.edu.pl

# S1. Method of data analysis

Water in a solution can be divided into two different contributions according to their properties, which result from interactions of solutes. Water with properties changed by a solute is called affected water (a). Bulk water (b), whose properties are not modified by the solute, can be also found in the solution. This kind of water is identical to pure water.

In Figure S1, different kinds of interactions, as the result of the interaction between solutes and their hydration sphere are presented. Water affected simultaneously by both solutes is called “double” affected water. Scenarios presented situations in Figure S1 in the paper are simplified schemes of real situations in solutions.

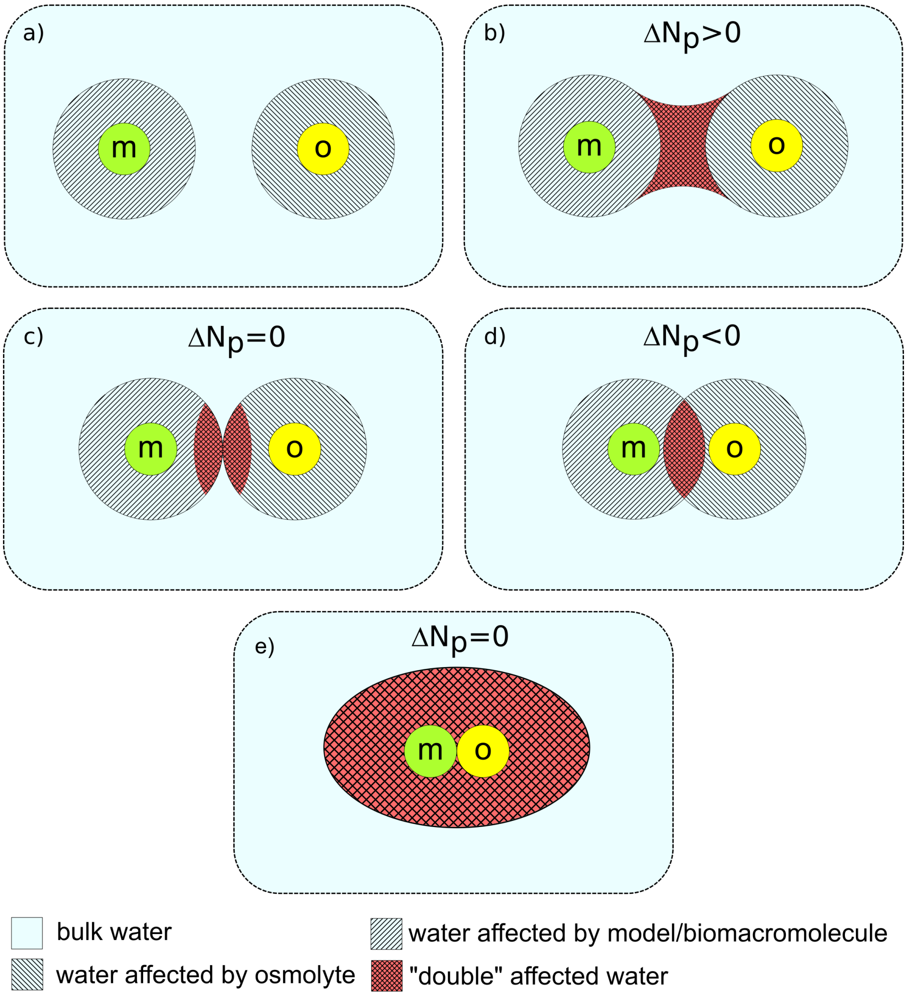


**Figure S1:** Boundary cases of mutual interactions of the hydration spheres of two different solutes in solution: a) hydration spheres of solutes do not interact with each other, b) hydration spheres of solutes cause the influence of additional water molecules from the bulk water which cross-linking these hydration spheres, c) hydration spheres of solutes do not overlap, but their direct proximity mutually influence water molecules within hydration spheres, d) overlapped hydration spheres of solutes, e) two different solutes interact with each other and their hydration spheres are modified forming the new hydration sphere. Scenarios a-d are taken from Ref. 1

## S1.1. Analysis of systems containing one solute

In a solution, which contains one kind of solute, the total molar concentration of water, $c$ [mol·dm$^{-3}$], can be expressed as $c = c_a + c_b$, where $c_a$ is affected water molar concentration and $c_b$ is bulk water molar concentration. A spectrum of affected water can be isolated with the use of the difference spectra method.[2] According to the premise of the method, the spectrum of affected water can be obtained with the following equation: $\varepsilon = (\varepsilon_a \cdot c_a + \varepsilon_b \cdot c_b)/c$, where $\varepsilon$ is the measured spectrum of water in the solution (in molar absorption scale) at molality $m$ [mol·kg$^{-1}$], $\varepsilon_a$ is affected water spectrum, $\varepsilon_b$ is bulk water spectrum. The rearrangement of the formula gives the affected water spectrum (7).

$$\varepsilon_a = \frac{c}{c_a}(\varepsilon - \varepsilon_b) + \varepsilon_b \tag{1}$$

The substitution $c_a = Nm/V$ for $c_a$ and $c_b = 1/MV$ for $c_b$ gives the following expression (2)

$$\varepsilon_a = \frac{1}{NMm}(\varepsilon - \varepsilon_b) + \varepsilon_b \tag{2}$$

where $N$ is affected number, which is equal to the number of moles of water molecules affected by one mole of solute, $M$ [kg·mol$^{-1}$] is the average molar mass of water (4% $D_2O$ in $H_2O$), $V$ is the volume of solution containing one kilogram of water. The procedure of finding the affected spectrum of water was illustrated in Figure S2 based on GLY solution in water. The measured spectra of $H_2O$ and mixture of $H_2O$ + $D_2O$ are shown in Figure S2a, while the corresponding spectra of $H_2O$ + GLY and $H_2O$ + $D_2O$ + GLY are presented in Figure S2b. The absorption of $H_2O$ has been eliminated by subtracting the absorption of the reference solution (without $D_2O$), recalculated for the same concentration of $H_2O$ as in the sample solution. This way spectra of pure HDO ($\varepsilon_b$) and HDO in a solution containing GLY ($\varepsilon$) were obtained (Figure S2c). The proper spectrum of affected water ($\varepsilon_a$) is found within a series of trial affected water spectra (some of them are shown in Figure S2d) obtained using Eq. 2 for different values of the $N$ parameter. Trial spectra were fitted with the use of analytical bands. The minimal number of analytical bands, which gives a sufficient fit, is the appropriate number of component bands. The product of Gaussian and Lorentzian peak functions is used as the starting analytical band shape. Parameters of the analytical bands (Gaussian to Lorentzian ratio, shape, intensity, half–width, and position) may vary during fitting. Then the spectrum of pure HDO was added to the decomposition of the spectrum containing analytical bands. That system was

fitted again. The parameters of the analytical band were unconstrained, while only the intensity of the pure water band remained variable during analysis. For us, the real affected water spectrum is the first spectrum in a series in which the spectral contribution of bulk water is less than 0.5%. With the real spectrum of affected water, $\varepsilon_a$, simultaneously the proper number o affected water molecules, $N$, is obtained.

At the stage of extracting the affected water spectrum, we treat analytical component bands as functions enabling approximation the band shape of the affected or the trial affected water, without attributing a physical sense to them. We attribute to them such a sense when required, usually supporting interpretation with theoretical calculations.

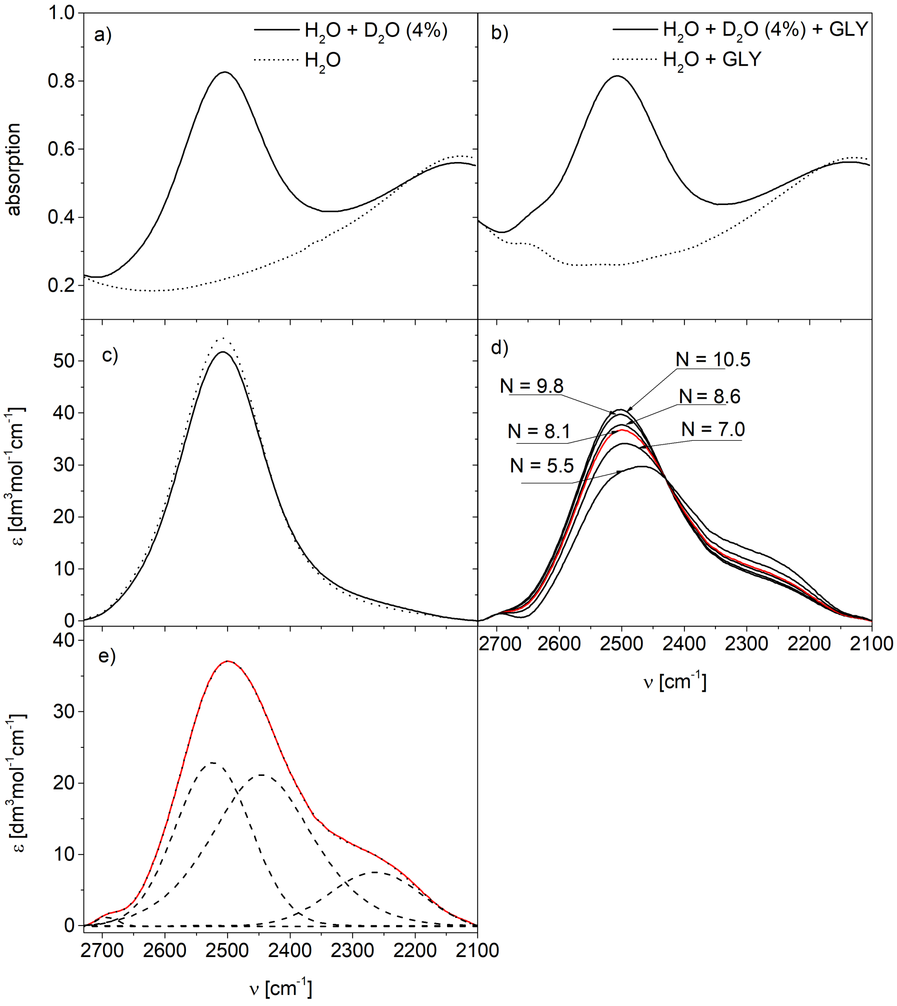


**Figure S2:** The procedure of finding the OD band of water affected by solute ($\varepsilon_a$) on the example of GLY. a) measured spectrum of $H_2O$ and $H_2O$ + $D_2O$ (4% $D_2O$ (by weight) in $H_2O$), b) measured spectrum of $H_2O$ + GLY and $H_2O$ + $D_2O$ (4%) + GLY c) spectrum of pure HDO ($\varepsilon_b$, dotted line) and spectrum of HDO in the solution containing GLY ($\varepsilon_b$, solid line), d) series of trial affected spectra (the proper affected spectrum is marked on red), e) decomposition of the affected spectrum: dashed line denotes analytical bands, dotted line denotes the sum of component bands, the solid red line denotes original affected spectrum.

## S1.2. Analysis of systems containing two solutes

A hypothetical situation of two not interacting solutes and their hydration spheres can be seen in Figure 1a. The only affected water populations in such solutions are those in the vicinity of solutes. The spectrum of this kind of solution, $\varepsilon_s$, is constructed from the spectra of solutions containing one solute with the use of the expression (3) [1]. This spectrum is later referred as "synthetic" spectrum of affected water:

$$\varepsilon_s = \frac{\varepsilon_{a_m} + \beta \cdot \varepsilon_{a_o} \cdot N_o/N_m}{\beta \cdot N_o/N_m + 1} \tag{3}$$

where $\varepsilon_{a_m}$ and $\varepsilon_{a_o}$ are the spectra of affected water in solutions containing only osmolyte ($o$) or only model substance/biomacromolecule ($m$). $N_m$ and $N_o$ are the number of affected water molecules in $m$ or $o$ in these aqueous solutions, $\beta$ is the molar ratio of $o$ to $m$ in a ternary solution. For the $\varepsilon_s$ spectrum a $N_s$ parameter may be determined (4). It denotes the number of affected water molecules by a sum of $o$ + $m$ equal to one mole.

$$N_s = \frac{N_m + \beta \cdot N_o}{\beta + 1} \tag{4}$$

The spectrum of a real solution (Figure 1b-e), containing spectral contribution of all additional kinds of affected water, is called "experimental" affected water spectrum, $\varepsilon_e$. Such a spectrum can be found using the following expression (5).

$$\varepsilon_e = \frac{1}{N_e M m_e}(\varepsilon - \varepsilon_b) + \varepsilon_b \tag{5}$$

where: $N_e$ is the number of "experimental" affected water molecules, it represents the number of affected water molecules by a sum of moles of $m$ and $o$ equal to one ($n_m + n_o = 1$ mole); $m_e$ is the total molality of $o$ and $m$ in the solution ($m_e = m_o + m_m$).

To facilitate the comparison of the number of affected water molecules corresponding to the "experimental" ($N_e$) and "synthetic" ($N_s$) affected spectra, the values of the affected water molecules were transformed into the $N_p$ functions: $N_{p_e} = N_e \cdot (\beta + 1)$ or $N_{p_s} = N_s \cdot (\beta + 1)$. These functions present the situation for the set consist of 1 mole of a model molecule/biomacromolecule and $\beta$ moles of osmolyte. In our studies, we established that the error of the number of affected water molecules de-

[1]This equation has now been modified to adequately account for the different numbers of affected water for both solutes in the solution compare to Equation (4) from Ref. 1.

termination equals 5 %. This value was found based on analyses carried out for various initial parameters of analytical bands and different systems. This error translates into the $N$ value error equal to 0.5 for $N < 10$. Error bars for $N_p$ values in Figures 1 and 4 in the Manuscript come from adding errors for each mole of the affected molecule making a set consisting of 1 model molecule/biomacromolecule and $\beta$ osmolyte.

## S1.3. Isolation of the "double" affected water spectrum

The spectrum of "double" affected water ($\varepsilon_d$), can be isolated by subtracting the "synthetic" spectrum of affected water from the corresponding "experimental" spectrum of affected water (6):

$$\varepsilon_d = \frac{\varepsilon_e - k \cdot \varepsilon_s}{1 - k} \quad (6)$$

where $k$ is subtracting coefficient which can be expressed as $k = 1 - |N_d|/N_s$. The way of its determination for different mutual interactions of solutes is described in the following part of Supporting Information. The expression $\varepsilon_e - k \cdot \varepsilon_s$ in equation 6 is the spectral contribution of "double" affected water. Dividing this expression by $1 - k$ is a conversion to one mole of "double" affected water.

The interaction of hydration spheres of two different solutes, as a result of the presence of excess "double" affected water (i.e., cross-linking water), is presented in Figure 1b. Molecules of such water are incorporated from the bulk water. The number of "double" affected water, which cross-links molecules, can be expressed as $N_d = N_e - N_s$ ($N_d > 0$). The "double" affected water spectrum can be obtained by subtracting the "synthetic" spectrum of affected water from the "experimental" spectrum of affected water with $k$ equal to 1.

The interaction of hydration spheres of two different solutes when no overlapping occurs is shown in Figure 1c. As a result of the close vicinity of hydration spheres of two different solutes, they mutually affect the water inside them. However, such an interaction does not change the number of affected water molecules. A differentiation between this and other situations is possible by the comparison of "experimental" affected water spectrum ($N_e$) with the corresponding "synthetic" spectrum of affected water ($N_s$). In this case, the real "double" affected water spectrum is found in a series of trial spectra (Eq. 6) obtained for different $k$ parameter values. Next, the contribution of the "synthetic" affected water spectrum in these spectra is examined. The first spectrum containing less than 0.5% of "synthetic" affected water spectral contribution is considered as the proper "double" affected water

spectrum in this scenario of interactions.

Figure 1d provides an example of the overlapping of hydration spheres of two different solutes. As a result, some of the water molecules are shared between them and the other water molecules are freed to bulk water. In that case, $N_d$ value is negative. It simply means that the number of affected water molecules in the "experimental" system is smaller when compared to the corresponding "synthetic" system. In that case, $|N_d|$ represents the number o water molecules shared between hydration spheres of different solutes. The rest of the water molecules are "synthetic" affected water. Their spectral contributions need to be subtracted to isolate the spectrum of "double" affected. It can be done using equation 6.

It is possible that different solutes in the solution interact directly with each other (Figure 1e). As the result of this type of interactions, hydration spheres of two solutes can transform (re-shape) into a completely new hydration sphere with properties different than for the starting ones. In this borderline scenario, the resulting "experimental" affected water spectrum should not contain any contribution of corresponding "synthetic" water spectrum. If the spectral contribution of "synthetic" affected water is present the isolation of affected spectra should be carried by using procedure described for the scenario in Figure 1c.

For the GLY + TMG and NMG + TMG systems we were unable to determine the spectra contribution of "double" affected water with the described method, i.e. using an appropriate subtraction factor of the spectrum of "synthetic" affected water from the spectrum of the "experimental" affected water. In some regions of the "double" affected water spectra obtained that way were negative. This indicated a partial conversion of hydration spheres of solutes into a new hydration sphere (Figure 1e), due to formation a molecular complex between various solutes. In that case we used classical method of data analysis, based on the checking of spectral contributions of the "synthetic" water spectrum in a series of trial "double" affected water spectra, by analogy to the method of finding the spectrum of water affected by the presence of only one solute in a solution.[2]

## S1.4. Transformation of water spectra into O···O distance distribution function

The molar absorptivity band contour was transformed into the probability distribution of the intermolecular O···O distance, $P(R_{OO})$. The empirical relationship between $\nu_{OD}$ and $R_{OO}$ (7) determined

by Berglund *et al*[3] was used in this procedure. This relationship is based on the position of HDO bands in the solid hydrates, which was measured with the use of infrared spectroscopy, and the respective intermolecular distances determined by diffraction methods. This empirical relationship is in the form:

$$\nu_{OD} = 2727 - 8.97 \cdot 10^6 \cdot e^{-3.73 \cdot R_{OO}} \tag{7}$$

with $R_{OO}$ expressed in Å and $\nu_{OD}$ in cm$^{-1}$, 2727 [cm$^{-1}$] denotes the experimental $\nu_{OD}$ of HDO in the gas phase. Taking into account that $ln(8.97 \cdot 10^6) = 16.01$, it can be written as $\nu_{OD} = 2727\text{˘}e^{16.01-3.73 \cdot R_{OO}}$, and this form of the equation is used by us. The band contour is then transformed to the probability distribution by taking the derivative according to

$$P(R) = C\varepsilon(\nu_{OD})\frac{d\nu_{OD}}{dR_{OO}} \tag{8}$$

$$\int_0^\infty P(R_{OO})\, dR_{OO} = 1 \tag{9}$$

where the normalization constant $C$ is chosen such that the probability distribution is properly normalized to unity, and $\varepsilon(\nu_{OD})$ is the absorbance. We have implemented this procedure to GRAMS/32 software.

# S2. Solutes affected-water spectra

In our previous works,[4–6] we removed the spectral contribution of ND vibration bands in affected water spectra. However, the procedure of their removal was laborious and time-consuming. Yet applying this procedure did not change the interpretation of the isolated spectra. Therefore, we did not remove the contribution of ND bands in our recent work.[1] The spectra of affected water in *trpzip-1* or *hewl* systems may also contain a small spectral contribution of OH bands, substituted as OD.

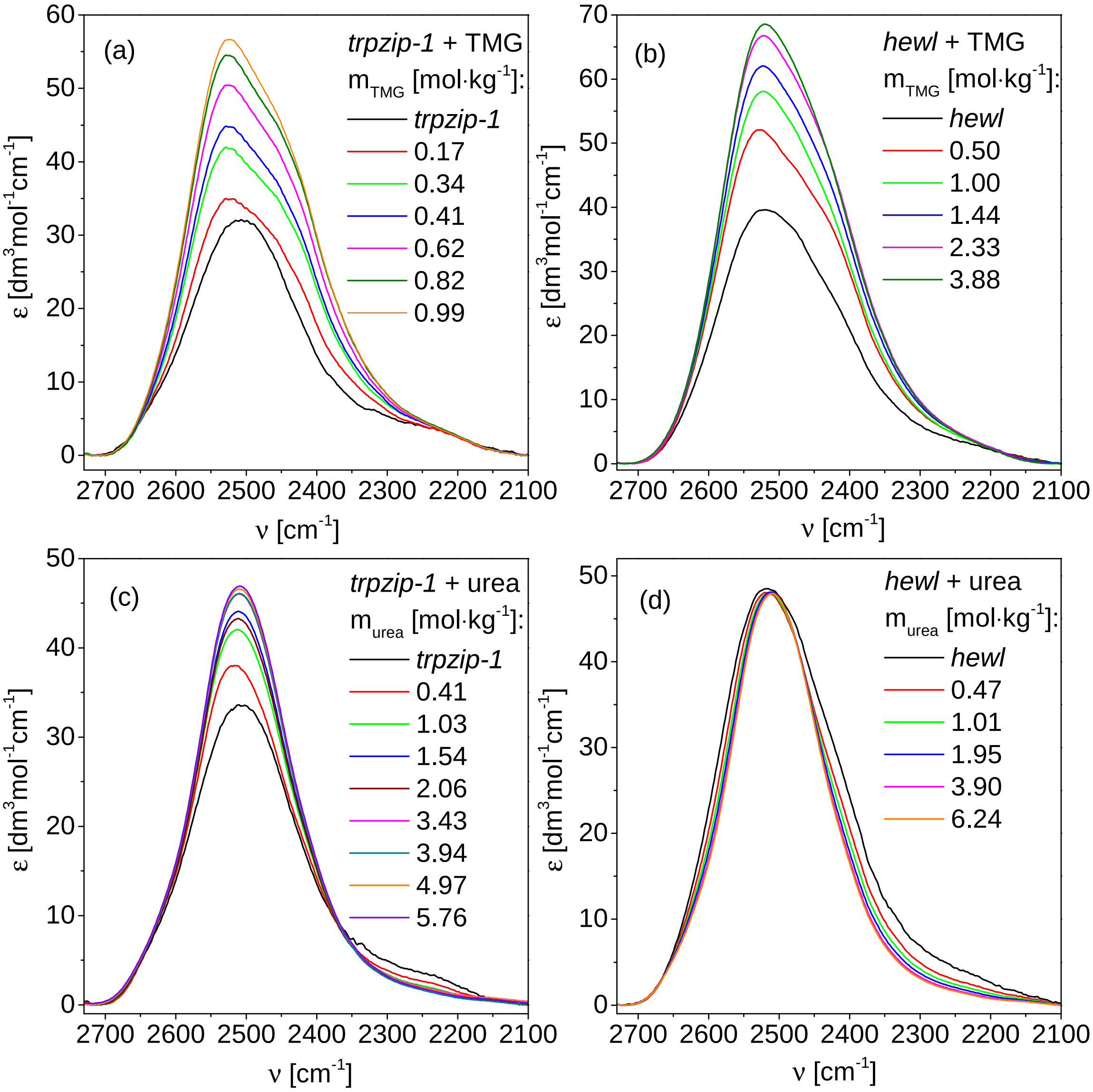


**Figure S3:** Spectra of "experimental" affected water for: a) *trpzip-1* + TMG, b) *hewl* + TMG, c) *trpzip-1* + urea, d) *hewl* + urea. The black line indicates the spectrum of water affected only by the biomacromolecule.

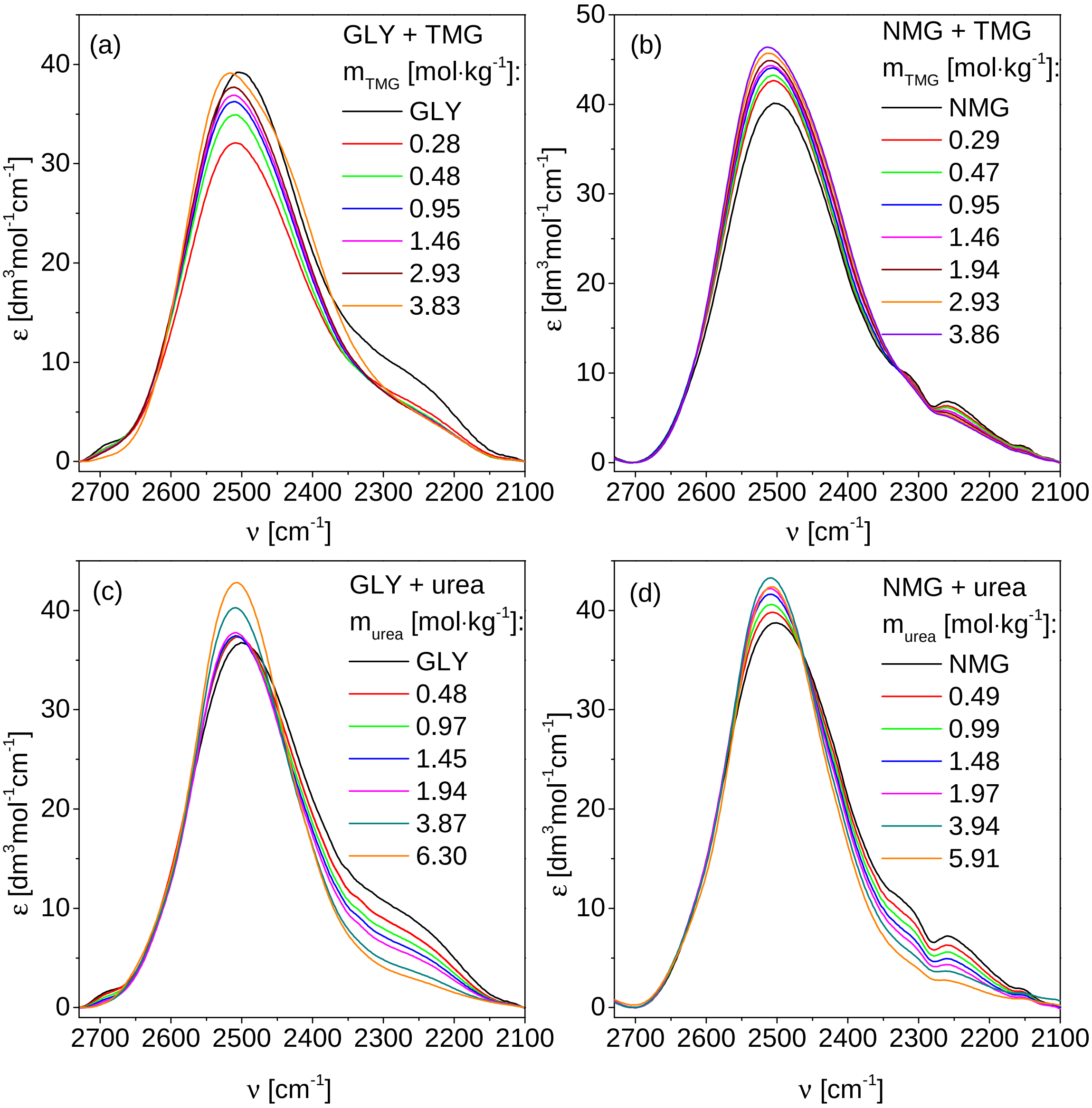


**Figure S4:** Spectra of "experimental" affected water for: a) GLY + TMG, b) NMG + TMG, c) GLY + urea, d) NMG + urea. The black line indicates the spectrum of water affected only by the model molecule.

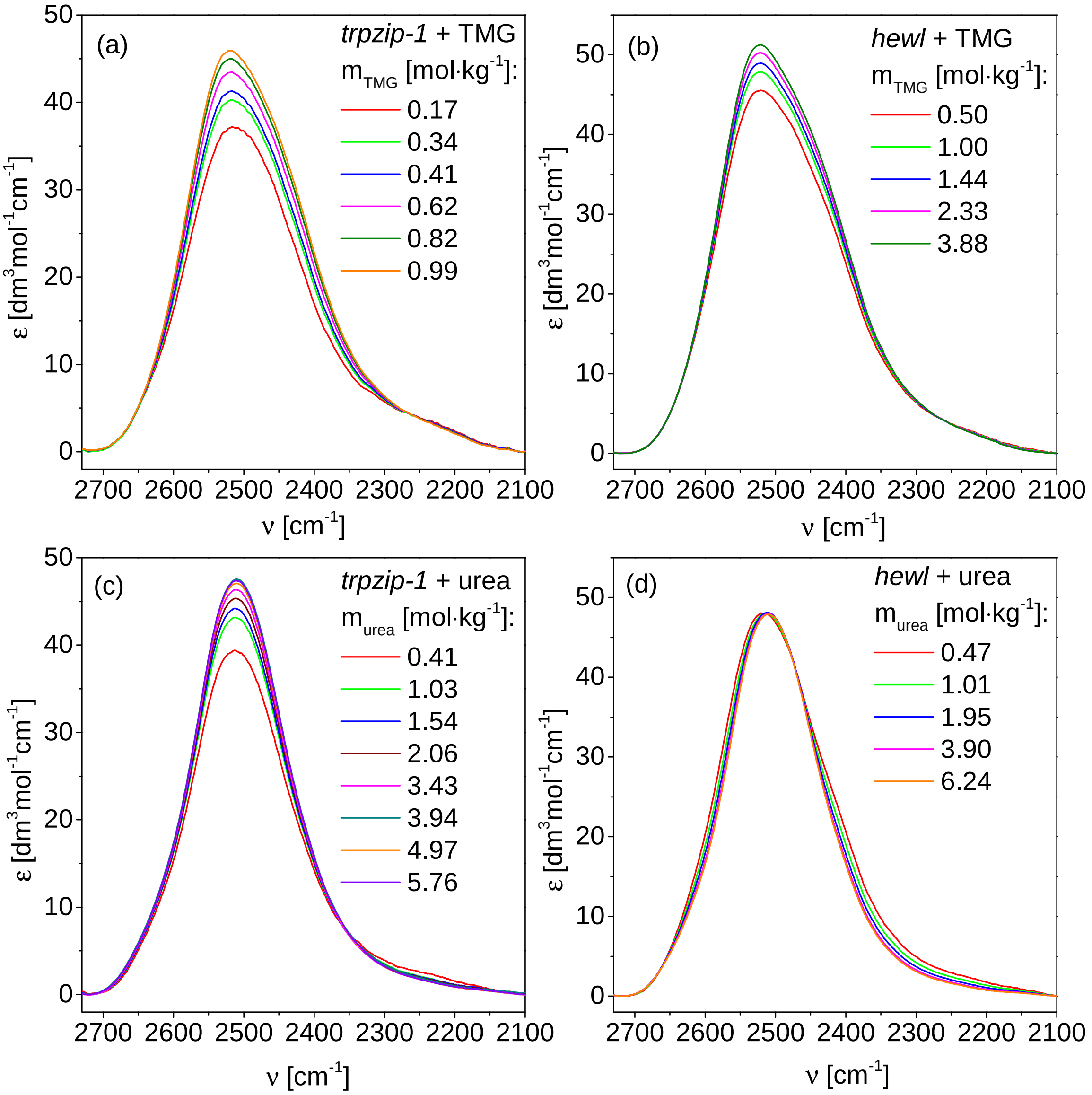


**Figure S5:** Spectra of "synthetic" affected water for: a) *trpzip-1* + TMG, b) *hewl* + TMG, c) *trpzip-1* + urea, d) *hewl* + urea.

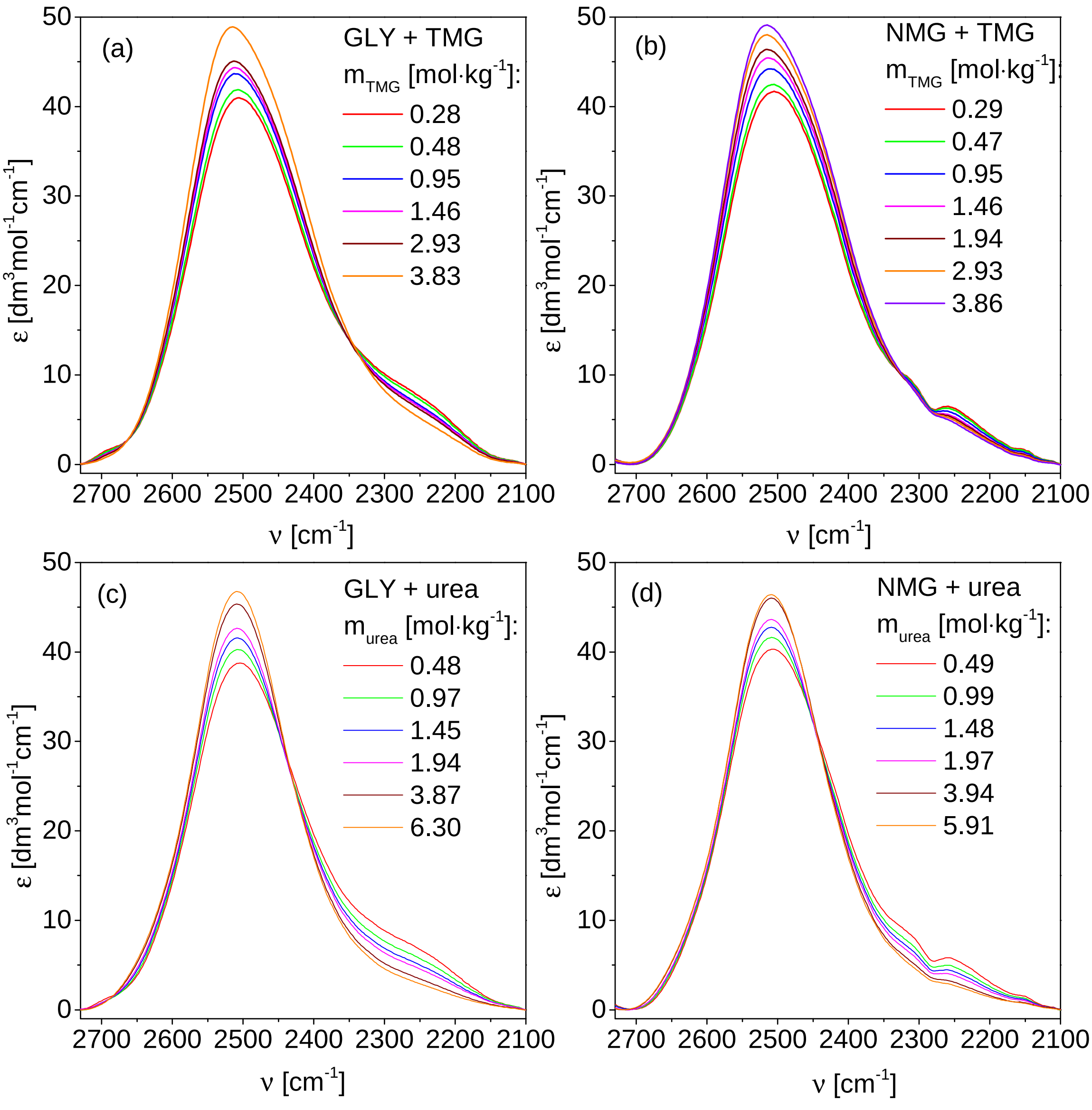


**Figure S6:** Spectra of "synthetic" affected water for: a) GLY + TMG, b) NMG + TMG, c) GLY + urea, d) NMG + urea.

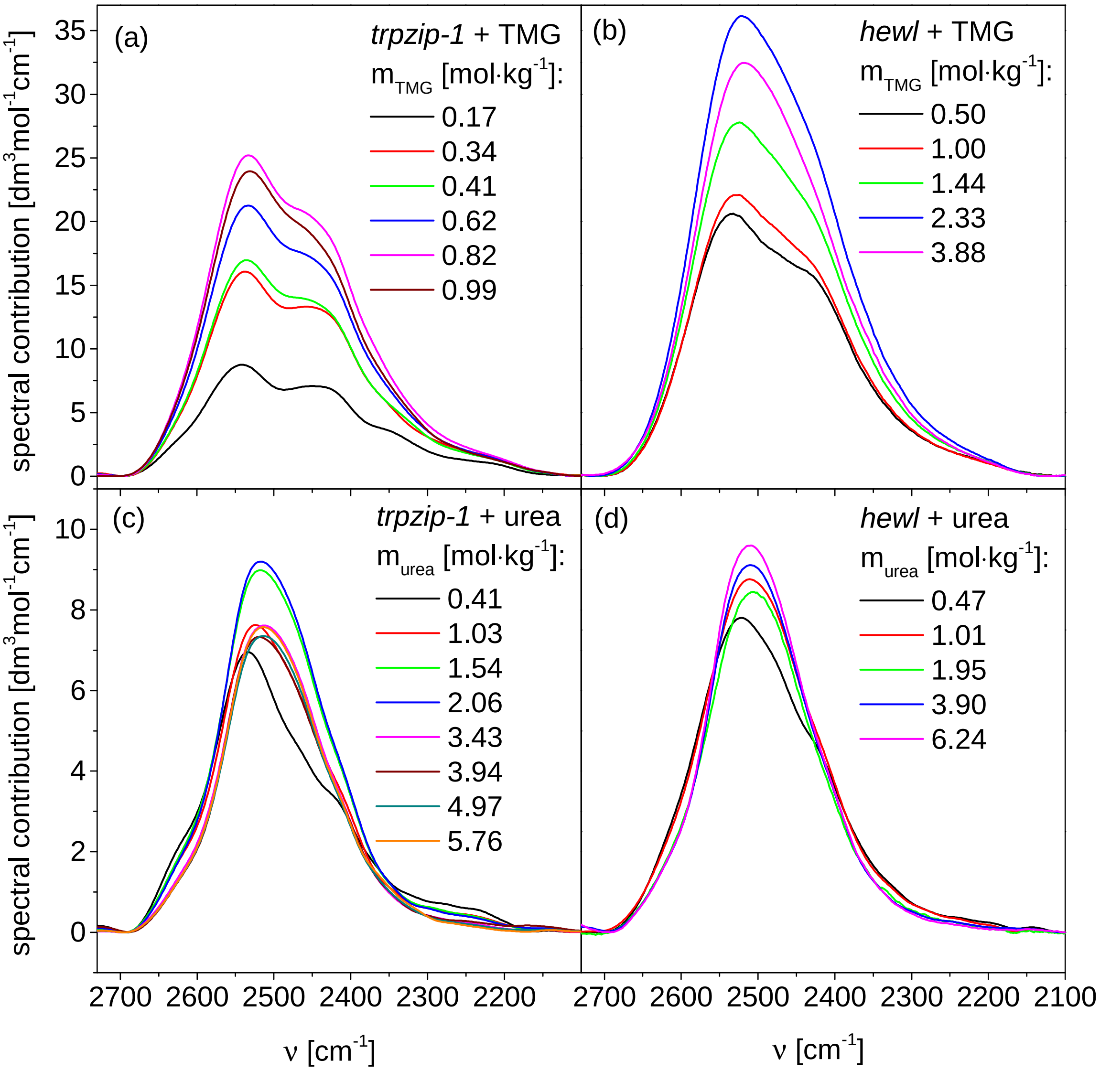


**Figure S7:** Spectral contribution of "double" affected water for: a) *trpzip-1* + TMG, b) *hewl* + TMG, c) *trpzip-1* + urea, d) *hewl* + urea.

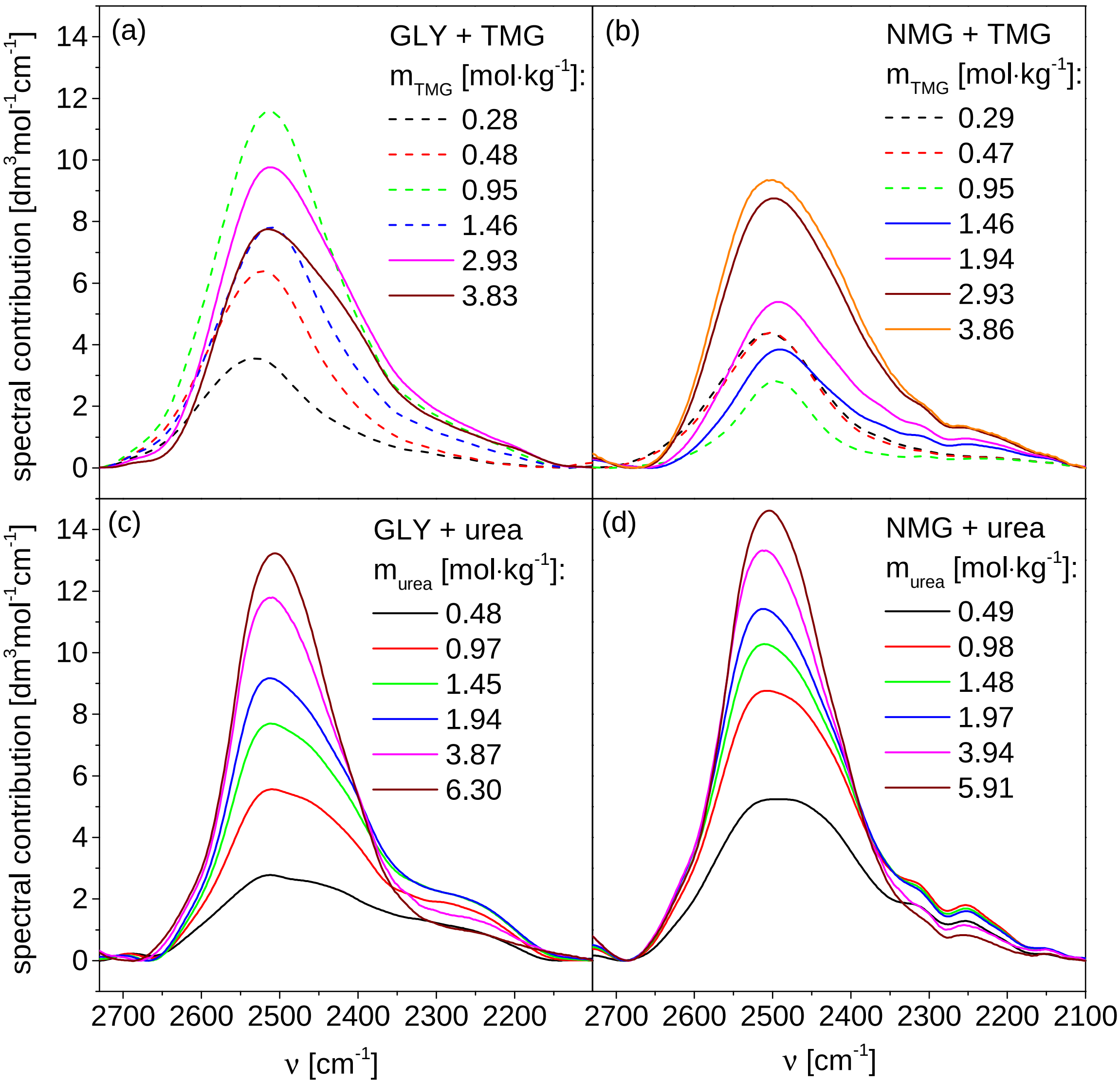


**Figure S8:** Spectral contribution of "double" affected water for: a) GLY + TMG b) NMG + TMG c) GLY + urea d) NMG + urea. The dashed line indicates cross-linking water, while the solid line indicates shared water.

**Table S1:** Difference in the number of affected water molecules obtained for "experimental", $N_{p_e}$, and "synthetic", $N_{p_s}$, affected water spectra $\Delta N_p = N_{p_e} - N_{p_s}$ as a function of molality of osmolyte, $m$, for model + osmolyte system.

| GLY + TMG | | NMG + TMG | | GLY + U | | NMG + U | |
|---|---|---|---|---|---|---|---|
| $m_{TMG}$ | $\Delta N_p$ | $m_{TMG}$ | $\Delta N_p$ | $m_{urea}$ | $\Delta N_p$ | $m_{urea}$ | $\Delta N_p$ |
| 0.28 | 2.7 | 0.29 | 0.6 | 0.48 | -1.2 | 0.49 | -1.5 |
| 0.48 | 3.1 | 0.47 | 0.6 | 0.97 | -2.8 | 0.99 | -3.1 |
| 0.95 | 3.0 | 0.95 | -0.1 | 1.45 | -4.6 | 1.48 | -4.2 |
| 1.96 | 0.9 | 1.49 | -1.6 | 1.94 | -6.1 | 1.97 | -5.4 |
| 2.93 | -1.2 | 1.94 | -2.2 | 3.87 | -10.8 | 3.94 | -10.2 |
| 3.83 | -2.6 | 2.93 | -4.0 | 6.30 | -15.6 | 5.91 | -16.0 |
| | | 3.86 | -4.3 | | | | |

**Table S2:** Difference in the number of affected water molecules obtained for "experimental", $N_{p_e}$, and "synthetic", $N_{p_s}$, affected water spectra $\Delta N_p = N_{p_e} - N_{p_s}$ as a function of molality of osmolyte, $m$, for biomacromolecule + osmolyte system.

| *trpzip-1* + TMG | | *hewl* + TMG | | *trpzip-1* + U | | *hewl* + U | |
|---|---|---|---|---|---|---|---|
| $m_{TMG}$ | $\Delta N_p$ | $m_{TMG}$ | $\Delta N_p$ | $m_{urea}$ | $\Delta N_p$ | $m_{urea}$ | $\Delta N_p$ |
| 0.17 | -25 | 0.50 | -186 | 0.41 | -26 | 0.47 | -99 |
| 0.34 | -38 | 1.00 | -215 | 1.03 | -45 | 1.01 | -173 |
| 0.41 | -38 | 1.44 | -371 | 1.54 | -68 | 1.95 | -279 |
| 0.62 | -47 | 2.33 | -611 | 2.06 | -88 | 3.90 | -605 |
| 0.82 | -59 | 3.88 | -771 | 3.43 | -104 | 6.24 | -996 |
| 0.99 | -55 | | | 3.94 | -122 | | |
| | | | | 4.63 | -132 | | |
| | | | | 5.76 | -165 | | |

**Table S3:** pH changes in function of osmolyte molality (mol·$kg^{-1}$) for biomacromolecule + osmolyte system. pH of pure *hewl* ($c = 104\ mg \cdot mL^{-1}$, ) and *trpzip-1* ($c = 54\ mg \cdot mL^{-1}$) were 4.93 and 1.92, respectively.

| *trpzip-1* + TMG | | *hewl* + TMG | | *trpzip-1* + urea | | *hewl* + urea | |
|---|---|---|---|---|---|---|---|
| $m_{TMG}$ | *pH* | $m_{TMG}$ | *pH* | $m_U$ | *pH* | $m_U$ | pH |
| 0.996 | 3.42 | 1.015 | 5.13 | 2.229 | 2.48 | 2.109 | 5.02 |
| | | 1.941 | 5.30 | 4.397 | 2.65 | 4.136 | 5.05 |
| | | 3.959 | 5.62 | 6.289 | 2.88 | 6.552 | 5.13 |

# References


(1) Stasiulewicz, M.; Panuszko, A.; Śmiechowski, M.; Bruździak, P.; Maszota, P.; Stangret, J. Effect of urea and glycine betaine on the hydration sphere of model molecules for the surface features of proteins. *Journal of Molecular Liquids* **2021**, *324*, 115090.

(2) Śmiechowski, M.; Stangret, J. Vibrational spectroscopy of semiheavy water (HDO) as a probe of solute hydration. *Pure and Applied Chemistry* **2010**, *82*, 1869.

(3) Berglund, B.; Lindgren, J.; Tegenfeldt, J. On the correlation between deuteron quadrupole coupling constants, O-H and O-D stretching frequencies and hydrogen-bond distances in solid hydrates. *Journal of Molecular Structure* **1978**, *43*, 179–191.

(4) Panuszko, A.; Bruździak, P.; Zielkiewicz, J.; Wyrzykowski, D.; Stangret, J. Effects of urea and trimethylamine-N-oxide on the properties of water and the secondary structure of hen egg white lysozyme. *Journal of Physical Chemistry B* **2009**, *113*, 14797.

(5) Panuszko, A.; Wojciechowski, M.; Bruździak, P.; Rakowska, P. W.; Stangret, J. Characteristics of hydration water around hen egg lysozyme as the protein model in aqueous solution. FTIR spectroscopy and molecular dynamics simulation. *Physical Chemistry Chemical Physics* **2012**, *14*, 15765.

(6) Panuszko, A.; Nowak, M. G.; Bruździak, P.; Stasiulewicz, M.; Stangret, J. Amides as models to study the hydration of proteins and peptides — spectroscopic and theoretical approach on hydration in various temperatures. *Journal of Molecular Liquids* **2019**, *278*, 706.
